# Improved quality of life, reduced quantitative lung fibrosis in a trial of inhaled pirfenidone for idiopathic pulmonary fibrosis

**DOI:** 10.1186/s12890-026-04234-x

**Published:** 2026-03-23

**Authors:** Grace Hyun J. Kim, Felix A. Woodhead, Deepthi Nair, Hao Bao, Howard M. Lazarus, Craig S. Conoscenti, Jonathan G. Goldin

**Affiliations:** 1 Department of Radiological Sciences, David Geffen School of Medicine at UCLA, Los Angeles, CA USA; 2 Avalyn Pharma Inc., Boston, MA USA; 3 Voiant, Los Angeles, CA USA

**Keywords:** Idiopathic pulmonary fibrosis, Interstitial lung disease, Lung function, Quality of life, HRCT, KBILD, Minimal clinically important difference, Inhaled pirfenidone

## Abstract

**Background:**

AP01, a novel formulation of inhaled pirfenidone, has demonstrated potential for improved efficacy and safety for idiopathic pulmonary fibrosis (IPF) vs. oral pirfenidone. A 72-week phase 1b study, ATLAS, suggested reliable correlations between structural changes revealed by high-resolution computed tomography (HRCT) and functional changes in forced vital capacity (FVC). Correlations between symptom improvement and structural changes are less established. Using ATLAS data, this study aims to investigate correlations between quantitative HRCT scores and changes in patient-reported outcomes (PROs) in AP01-treated patients with IPF.

**Methods:**

Patients (*N* = 91) received AP01 50 mg qd or 100 mg bid. FVC, quantitative lung fibrosis (QLF), and PRO measures including King’s Brief Interstitial Lung Disease questionnaire (KBILD) assessed functional, structural, and symptomatic changes over 48 weeks. Non-contrast HRCTs were performed at baseline and 24 weeks; improvement was defined as > 2% decrease in QLF score.

**Results:**

Among 69 patients with sufficient quality HRCT scans, similar proportions on both regimens exhibited QLF improvement (50 mg qd: 16%; 100 mg bid:16%) and QLF stabilisation (-2% ≤ ∆QLF ≤ 2%) (50 mg qd: 47%; 100 mg bid: 55%). 100-mg bid subjects with improved QLF exhibited increased mean KBILD total scores from baseline beginning week 8 (8 points) through week 48 (> 20 points) (minimal clinically important difference = 5 points).

**Conclusions:**

A strong and lasting association between structural and symptomatic improvements confirms functional changes with AP01 and supports evaluation of the 100_-_mg bid dose.

**Clinical trial registration:**

AustralianNew Zealand Clinical Trials Registry Identifier ACTRN 12,618,001,838,202 URL: https://www.anzctr.org.au the trial registration took place before the first patient enrolled trial registration was 12 November 2018 and first patient enrolled on 06 January 2019.

**Supplementary Information:**

The online version contains supplementary material available at 10.1186/s12890-026-04234-x.

## Background

Idiopathic pulmonary fibrosis (IPF) is a fatal lung disease occurring primarily in older adults, characterised by a chronic course and a generally poor prognosis. It is a specific form of chronic fibrosing interstitial pneumonia limited to the lung and associated with the pathological pattern of usual interstitial pneumonia (UIP). IPF follows a variable clinical course in which periods of relative stability are mixed with episodes of accelerated decline, with a median survival of 3 to 5 years [1]. IPF can be treated with either pirfenidone or nintedanib, which have been shown to decrease the rate of forced vital capacity (FVC) decline in IPF patients compared with placebo [2]. However, both oral pirfenidone and nintedanib have challenging side effects that can limit their use. Therefore, new, better-tolerated therapies with comparable or improved efficacy continue to be needed in this patient population.

Inhalation therapy holds promise as a possible treatment for IPF. In general, inhaled formulations improve safety and efficacy by reducing systemic exposure and improving delivery to the lung tissue [3]. AP01 is a novel form of pirfenidone optimised for delivery by inhalation through a specially designed PARI investigational eFlow nebuliser (eFlow). AP01 phase 1 data showed that 100 mg of nebulised AP01 achieved a peak alveolar concentration 35 times that reached with the licensed oral dose of pirfenidone (801 mg three times daily), with ~ 1/15 the plasma level [4]. AP01 was well tolerated by normal healthy volunteers and IPF patients.

Safety and therapeutic effect of AP01 in the IPF population were assessed in ATLAS, a phase 1b, randomised, open-label, dose-response study. Patients were randomised at 25 sites to receive one of two doses of AP01, 50 mg once daily (qd) or 100 mg twice a day (bid) [5]. Both doses were well tolerated, and side effects associated with oral pirfenidone were notably less frequent, consistent with the reduced systemic exposure. The primary efficacy endpoint, change from baseline in FVC % predicted, remained stable in the 100-mg bid group. Further, symptoms as assessed by patient-reported outcomes (PROs), including the King’s Brief Interstitial Lung Disease (KBILD) questionnaire and the Leicester Cough Questionnaire (LCQ), remained stable or showed improvement. Change from baseline in FVC correlated with quantitative computed tomography (QCT) measures of lung fibrosis in patients receiving 100 mg bid AP01 [6]. 

Quantitative CT offers the potential to measure disease extent and change over time, which may be useful as a prognostic and outcome biomarker [7–9]. Several phase 2 and phase 3 clinical studies have explored using QCT measures of lung fibrosis [10–12]. FVC, a measure of function, is the most widely accepted primary endpoint for IPF trials. FVC and QCT, a measure of structure, have been shown to correlate in several studies, including ATLAS. Beyond function and structure, patients, clinicians, and regulators have called for the consideration of additional endpoints that better reflect patient experience, including multifactorial endpoints based on how the patient “feels, functions or survives,” together with more accessible PROs [6, 7]. 

Here, we report detailed quantitative high-resolution computed tomography (HRCT) results from the 72-week ATLAS trial with AP01 50 mg qd or 100 mg bid, as well as post-hoc analyses investigating the correlation of structure and symptom improvements as a measure of patient experience in IPF.

## Methods

### Study design and population

**Fig. 1:**
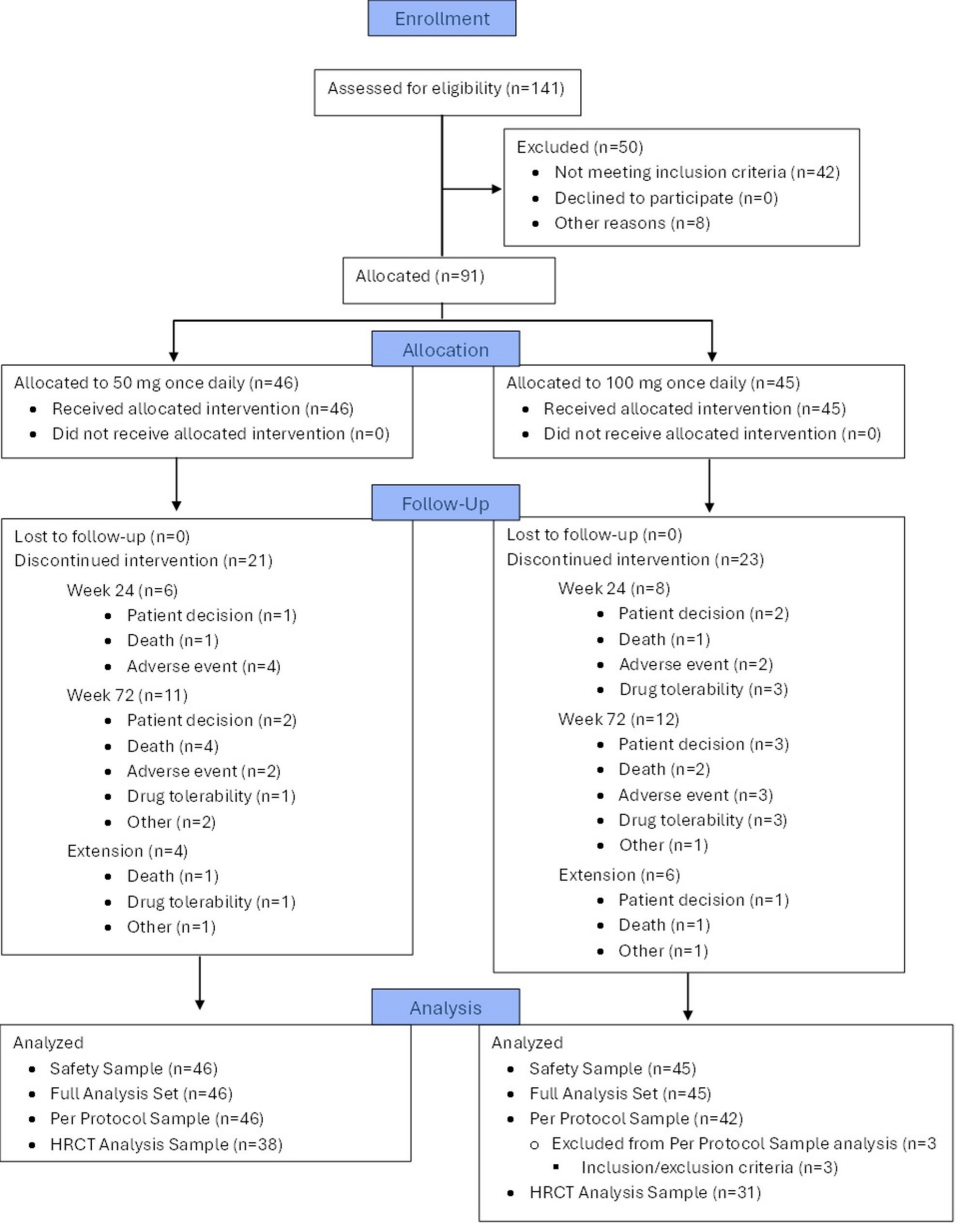
CONSORT flowchart for the ATLAS study

The phase 1b, randomised, open-label, dose-response, multicenter ATLAS study was conducted at 25 sites in six countries from May 2019 to October 2021 (ANZCTR: ACTRN 12618001838202) [5]. Patients were randomised in a 1:1 ratio to receive either AP01 50 mg qd or 100 mg bid (for 24 weeks, with a possible extension to 72 weeks). AP01 was administered with an investigational eFlow^®^ nebuliser system (PARI Pharma GmbH). The CONSORT flow diagram of this study is presented in Fig. 1.

This study was conducted in compliance with the principles of the Declaration of Helsinki and the Harmonised Tripartite Guideline for Good Clinical Practice of the International Council for Harmonisation. Local ethics committees/institutional review boards approved the protocol for each site, and all patients provided written informed consent before enrolment. Safety results were reviewed by a Data and Safety Monitoring Board.

Eligible patients were adults aged 40 years or older diagnosed with IPF within 5 years meeting the following criteria: 1) FVC 40%-90% predicted measured by spirometry; and 2) intolerant of, unwilling to take, or ineligible for oral pirfenidone or nintedanib; complete inclusion/exclusion criteria published previously [5]. 

The diagnosis of IPF was defined in accordance with the American Thoracic Society (ATS)/European Respiratory Society (ERS) clinical practice guideline [1]. Per ATS/ERS guidance, an available HRCT chest scan, obtained from the patient’s provider within the past 12 months or performed during screening, or a surgical lung biopsy was evaluated by site investigators. The HRCT scans were reviewed at sites to confirm the diagnosis of IPF by demonstrating the presence of definite or probable UIP pattern. The previously published quantitative lung fibrosis (QLF) algorithm was used to quantitate the extent of pulmonary fibrosis [13]. 

### Outcomes and data collection

Efficacy outcome measures in ATLAS included the absolute change in FVC % predicted (primary endpoint) and PRO scores from baseline to week 24 [5]. Prespecified secondary efficacy endpoints included the change from baseline in extent of fibrosis and lung volumes as measured by HRCT. Prespecified exploratory outcomes included correlation of the extent of fibrosis and lung volumes, as measured by HRCT at baseline and 24 weeks with spirometry.

Thin section non-contrast volumetric chest HRCT scans were performed at baseline on all subjects unless they had had an acceptable scan within the preceding month. A follow-up CT was originally scheduled at 24 weeks, but because of coronavirus disease 2019 (COVID-19) pandemic restrictions, this scan could be rescheduled for the next visit at 36 weeks. All follow-up HRCT scans are referred to hereafter as the 24-week scan. The QLF computer-aided score was expressed both as a percentage of pixels and in milliliters (mL) for the whole lung and each lobe, and the total lung capacity (TLC) volume in liters (L). The extent can be expressed in two scales of the percentage of involvement (%) or volume (mL) by multiplying the extent of disease in the percent scale and lung volume from HRCT [13, 14]. All HRCT personnel were blinded to treatment assignment.

PRO scores included the KBILD questionnaire, which was collected every four weeks for 24 weeks, then every 12 weeks through week 72 [5, 15, 16]. The KBILD questionnaire is validated for use in ILD patients [16]. It comprises questions relating to “breathlessness and activities,” chest symptoms, and psychological factors; each of these domains, as well as the total, has a scoring range of 0 to 100, with 100 being the best health status. The minimally clinically important difference (MCID) in total KBILD has been reported to be 5 points [17]. 

### Statistical analysis

Sample size calculations have been published previously [5]. 

For the analyses reported here, demographic and disease characteristics at baseline were summarised by dose group for patients with both a baseline and 24-week scan. QLF, quantitative ground glass (QGG), quantitative honeycomb (QHC), and TLC at baseline and 24 weeks, as well as absolute change from baseline to 24 weeks, were calculated and summarised descriptively by dose group.

A linear model was used to examine differences between dose groups in the change from baseline in whole-lung QLF from baseline to 24 weeks. Change from baseline was the dependent variable, and the dose group was the predictor variable. Baseline whole-lung QLF, geographic region, age at screening, sex, baseline diffusing capacity to carbon monoxide % predicted, and baseline progression-related biomarker values (CXCL13, CCL18, and MMP3, each categorised as > median level vs. ≤ median level), as well as time of the HRCT scan (relative to baseline in weeks), were considered for adjustment as covariates. Disease progression was categorised by change from baseline in QLF as a pre-defined percentage of the whole lung (improved: < -2%; stable: ≥ -2% and ≤ 2%, worsened: > 2%) [12]. Mean changes from baseline in KBILD total and domain scores were summarised and plotted over time by QLF progression category for each dose group to examine the relationship between quality of life (QOL) and disease progression. Missing data were not imputed. All analyses were conducted using SAS Version 9.4 (SAS Institute Inc., USA).

## Results

All 91 patients randomised had a baseline HRCT scan collected, and 94.5% (86/91) passed image quality review for quantitative imaging analyses. At 24 weeks, 77 patients had follow-up. A 24-week HRCT scan was collected for 91% (70/77) patients, of which 98.6% (69/70) were performed per protocol. Thus, 69 patients had both a baseline and 24-week HRCT scan available (i.e., patients with paired HRCT scans) for analysis, including 38 patients in the 50-mg qd group and 31 in the 100-mg bid group. In these 69 patients, 47 HRCT scans were performed at 24 weeks, 19 were delayed until 36 weeks because of COVID-19 restrictions, and 3 were performed early (12, 16, and 20 weeks) because of termination from the study. The results of analyses conducted in these 69 patients with paired HRCT scans are reported here. 

Demographics and baseline characteristics were similarly distributed in both dose groups, except HRCT pattern and QLF at baseline (Table 1). The 50-mg qd group had a smaller percentage of patients with a typical UIP pattern and greater fibrosis, both in mL and as a percentage of the whole lung, compared with the 100-mg bid group.

**Table 1:** Characteristics of patients with baseline and a 24-week HRCT scans by dose group

Characteristic	50 mg qd (*N* = 38)	100 mg bid (*N* = 31)	Total (*N* = 69)
Region, n (%)			
Asia Pacific	20 (52.6)	16 (51.6)	36 (52.2)
Europe	18 (47.4)	15 (48.4)	33 (47.8)
Age at screening (years), mean (SD)	73.4 (70.0)	70.7 (8.5)	72.2 (7.8)
Male, n (%)	28 (73.7)	24 (77.4)	52 (75.4)
White Race, n (%)	37 (97.4)	29 (93.5)	66 (95.7)
Former smoker, n (%)	27 (71.1)	22 (71.0)	49 (71.0)
Spirometry			
FVC % predicted at screening ≥ 50% predicted, n (%)	35 (92.1)	31 (100.0)	66 (95.7)
FVC at baseline, mean (SD)			
L	2.5 (0.59)	2.7 (0.64)	2.6 (0.62)
% predicted	69.8 (12.04)	73.2 (9.03)	71.3 (10.85)
FEV_1_% predicted at baseline, mean (SD)	76.2 (12.65)	77.4 (9.80)	76.8 (11.39)
D_LCO_ % predicted at baseline (mL/min/mm Hg), mean (SD)	47.2 (13.36)	48.3 (11.01)	47.7 (12.28)
HRCT			
Pattern, n (%)			
Typical UIP	14 (36.8)	17 (54.8)	31 (44.9)
Probable UIP	23 (60.5)	12 (38.7)	35 (50.7)
Indeterminate UIP	1 (2.6)	2 (6.5)	3 (4.3)
QLF at baseline, mean (SD)			
mL of whole lung	645.6 (307.52)	478.7 (304.25)	570.6 (315.10)
% of whole lung	18.4 (11.02)	11.9 (7.89)	15.5 (10.21)
Ground glass at baseline, mean (SD)			
mL of whole lung	729.9 (311.68)	727.6 (258.60)	728.8 (286.99)
% of whole lung	19.2 (6.53)	17.7 (6.10)	18.6 (6.34)
Honeycomb at baseline, mean (SD)			
mL of whole lung	22.7 (31.16)	20.2 (21.94)	21.6 (27.24)
% of whole lung	0.6 (0.89)	0.5 (0.56)	0.6 (0.76)
Total lung capacity (L), mean (SD)	3.8 (0.90)	4.2 (0.89)	4 (0.91)
KBILD score at baseline, mean (SD)			
Total	53.8 (13.54)	56.5 (11.82)	55.0 (12.78)
Breathlessness and activities	40.6 (16.97)	43.0 (19.94)	41.7 (18.27)
Chest symptoms	64.7 (23.73)	69.8 (21.57)	67.0 (22.76)
Psychological	53.8 (18.50)	57.7 (15.93)	55.6 (17.37)

All 24-week HRCT scan statistics included patients with an HCRT scan performed at 24 weeks, at 36 weeks (a rescheduled 24-week visit because of the coronavirus disease 2019 pandemic), or during the End of Treatment visit

*bid* Twice a day, *D*_*LCO*_Diffusing capacity to carbon monoxide, *FEV*_*1*_Forced expiratory volume in 1 s, *FVC* Forced vital capacity, *HRCT* High-resolution computed tomography, *KBILD* King’s Brief Interstitial Lung Disease questionnaire, *qd* Once daily, *QLF* Quantitative lung fibrosis, *UIP* Usual interstitial pneumonia

### Structural changes (via HRCT parameters) 

Table 2 displays descriptive summaries of absolute and % changes from baseline at 24 weeks in whole-lung HRCT parameters by dose group. The mean change from baseline in QLF showed a larger increase (i.e., less disease stabilisation) in the 50-mg qd group than in the 100-mg bid group. Analyses of the change in TLC were consistent with these results, showing a larger decrease in the 50-mg qd group. The adjusted, least-squares mean change from baseline in QLF was + 25.7 mL in the 50-mg qd dose group and − 29.5 mL in the 100-mg bid dose group, with a difference (100 mg bid – 50 mg qd) of -55.2 mL (95% CI: -145.6 to 35.2 mL). On average, there appears to be a smaller increase in QLF and a larger reduction in QGG in the 100-mg bid group than in the 50-mg qd group, whereas the latter showed better stability on QHC.

**Table 2:** Absolute and change from baseline in whole-lung HRCT parameters by dose group

Parameter, mean (SD)	50 mg qd (*N* = 38)	100 mg bid (*N* = 31)	Total (*N* = 69)
QLF			
mL			
Week 24	665.9 (331.29)	483.3 (317.36)	583.9 (335.45)
Change from baseline	20.3 (171.24)	4.6 (160.58)	13.3 (165.51)
%			
Week 24	19.3 (11.69)	12.4 (9.23)	16.2 (11.14)
Change from baseline	1.0 (6.15)	0.5 (4.55)	0.7 (5.46)
Quantitative ground glass			
mL			
Week 24	710.3 (298.61)	680.2 (245.57)	696.8 (274.50)
Change from baseline	-19.6 (112.24)	-47.2 (127.15)	-32.0 (119.07)
%			
Week 24	19.5 (7.04)	16.9 (6.66)	18.3 (6.94)
Change from baseline	0.2 (4.49)	-0.8 (4.14)	-0.2 (4.33)
Honeycomb			
mL			
Week 24	18.4 (24.10)	28.6 (52.06)	23.0 (39.21)
Change from baseline	-4.3 (21.94)	8.3 (41.67)	1.4 (32.68)
%			
Week 24	0.5 (0.68)	0.6 (0.96)	0.6 (0.81)
Change from baseline	-0.1 (0.59)	0.1 (0.90)	0.0 (0.75)
Total lung capacity			
Week 24 (L)	3.7 (0.85)	4.2 (0.98)	3.9 (0.93)
Change from baseline at week 24 (mL)	-114.2 (480.05)	-44.0 (326.51)	-82.7 (416.72)

*bid* Twice a day, *HRCT* High-resolution computed tomography, *qd* Once daily, *QLF* Quantitative lung fibrosis, *SD* Standard deviation

**Fig. 2:**
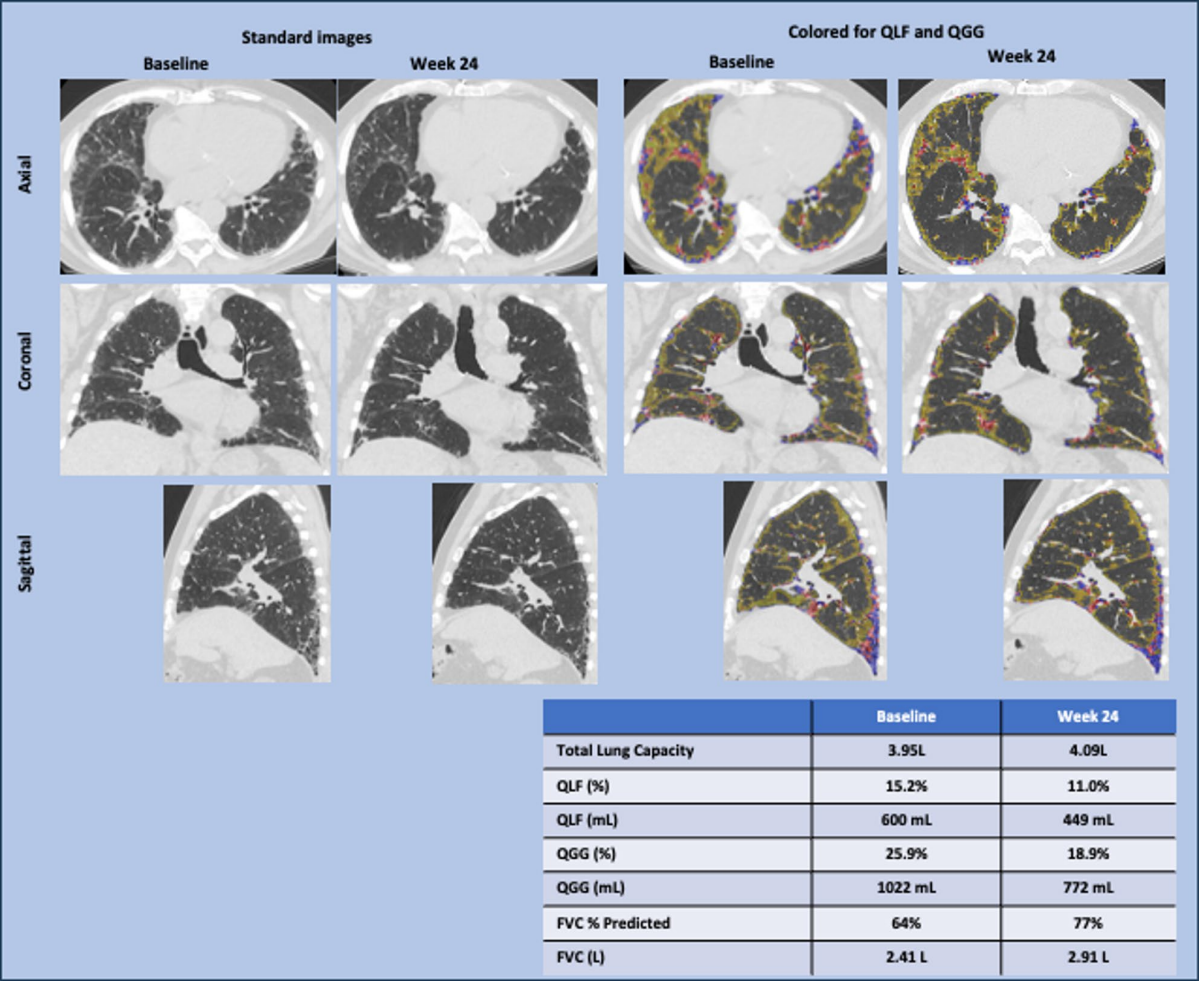
Baseline and week 24 images of a 100-mg bid patient showing improved QLF and FVC. bid = twice a day; FVC = forced vital capacity; HRCT = high-resonance computed tomography; QLF = quantitative lung fibrosis; QGG = quantitative ground glass. The images in the left two columns are the standard axial, coronal, and sagittal HRCT images for an individual patient treated with AP01 100 mg bid. The images in the right two columns are the overlaid qualitative results of the corresponding images. Blue dots indicate the results of QLF classification; yellow dots indicate results for QGG

The quantitative evaluation of HRCT for a representative individual patient in the 100-mg bid dose group is shown in Fig. 2, with the basis for QLF and QGG indicated by blue and yellow dots, respectively. This patient’s QLF score improved by 151 mL from a baseline value of 600 mL (15.2%) to 449 mL (11.0%), and the QGG score improved by 250 mL from a baseline value of 1022 mL (25.9%) to 772 mL (18.9%). TLC increased correspondingly from 3.95 L at baseline to 4.09 L at 24 weeks.

**Fig. 3:**
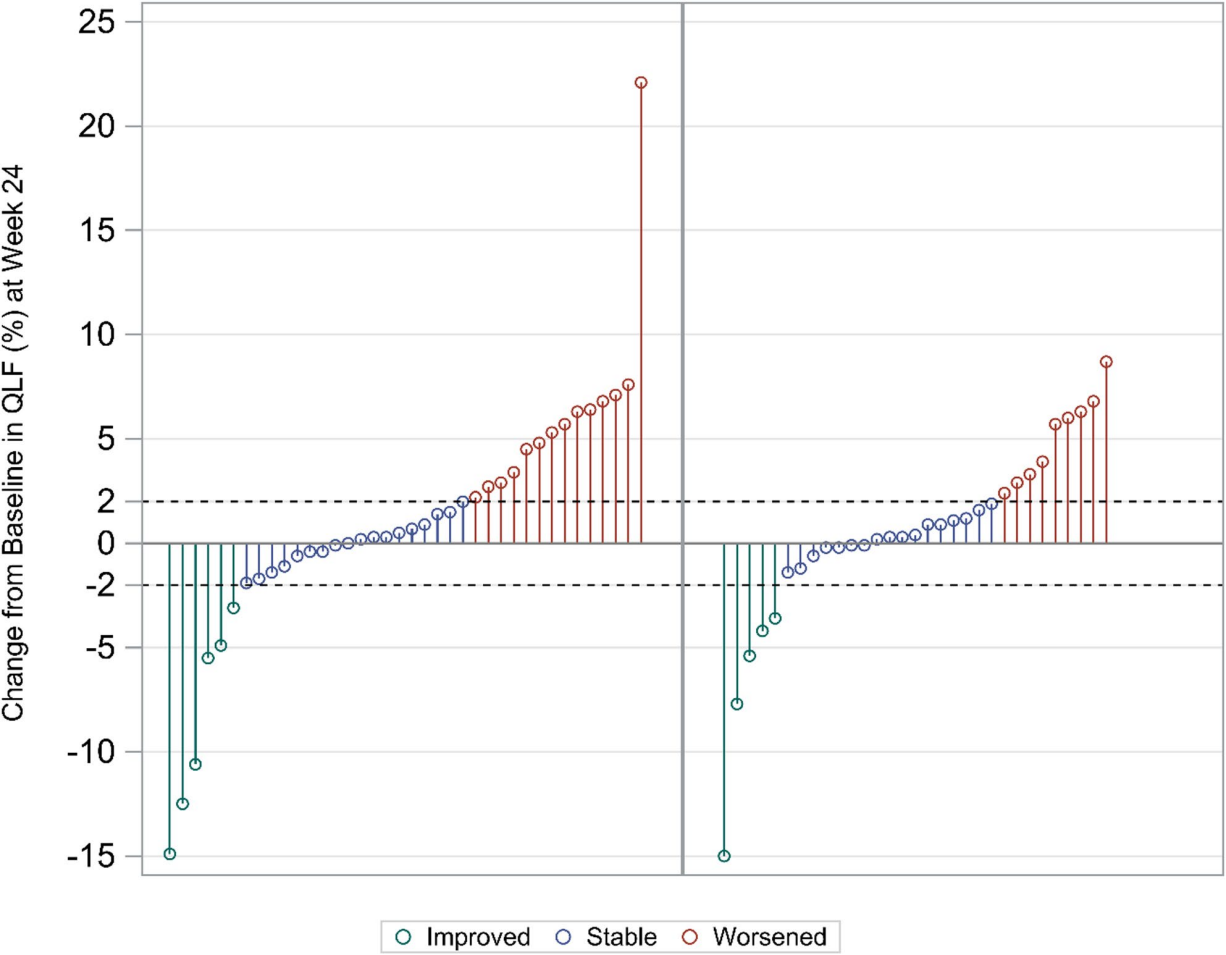
Waterfall plot of change in QLF at week 24 as a percentage of whole lung by 50 mg qd (left) and 100 mg bid (right), bid = twice per day; qd = once per day; QLF = quantitative lung fibrosis

Individual changes from baseline in QLF (%) at 24 weeks are illustrated in Fig. 3. The proportion of patients who improved (∆QLF < -2%) was similar (16%) in both dose groups. Stabilisation (-2% ≤ ∆QLF ≤ 2%) was achieved in a larger percentage of patients in the 100-mg bid group. Overall, 22 (71%) patients improved or stabilised in the 100-mg bid group vs. 24 (63%) in the 50-mg qd group.

### Correlations: structure / symptom improvements

Cross-classification of patients by symptom improvement, assessed by KBILD, and fibrosis improvement, assessed by QLF, shows the extent of agreement of these measures. Table 3 presents categorical summaries of KBILD improvement, with improvement defined as change from baseline ≥ MCID, and by QLF improvement category, where fibrosis improvement is defined as QLF change from baseline < -2%. In the 100-mg bid treatment groups, the majority (80%-100%) of patients with improved QLF scores had improved KBILD scores at week 24. Furthermore, the majority (63%-67%) of patients who had stable/worse QLF scores did not show improvement in KBILD. The pattern was less pronounced in the 50-mg qd treatment group.

**Table 3:** KBILD improvement from baseline by HRCT improvement from baseline

	KBILD score change from baseline at week 24							
	Total score		B & A score		Chest symptom score		Psychological score	
	< 5	≥ 5	< 5	≥ 5	< 5	≥ 5	< 5	≥ 5
	50 mg qd (*N*=37^a^)							
Improved^b^ QLF at week 24 (n = 6)	3 (50%)	3 (50%)	5 (83.3%)	1 (16.7%)	2 (33.3%)	4 (66.7%)	2 (33.3%)	4 (66.7%)
Stable/worse^c^ QLF at week 24 (*n* = 31)	27 (87.1%)	4 (12.9%)	26 (83.9%)	5 (16.1%)	20 (64.5%)	11 (35.5%)	22 (71.0%)	9 (29.0%)
	100 mg bid (*N* = 31)							
Improved^b^ QLF at week 24 (*n* = 5)	1 (20%)	4 (80%)	0	5 (100%)	2 (40%)	3 (60%)	1 (20%)	4 (80%)
Stable/worse^c^ QLF at week 24 (*n* = 24)	16 (66.7%)	8 (33.3%)	15 (62.5%)	9 (37.5%)	15 (62.5%)	9 (37.5%)	16 (66.7%)	8 (33.3%)

*B & A* Breathlessness and activities, *bid* Twice a day, *KBILD* King’s Brief Interstitial Lung Disease questionnaire, *qd* Once daily, *QLF* Quantitative lung fibrosis

^a^Analysis includes patients with week 24 data

^b^QLF improvement defined as < -2.0% change from baseline

^c^QLF stable and worse defined as ≥ -2.0 to ≤ 2% and> 2%, respectively

**Fig. 4:**
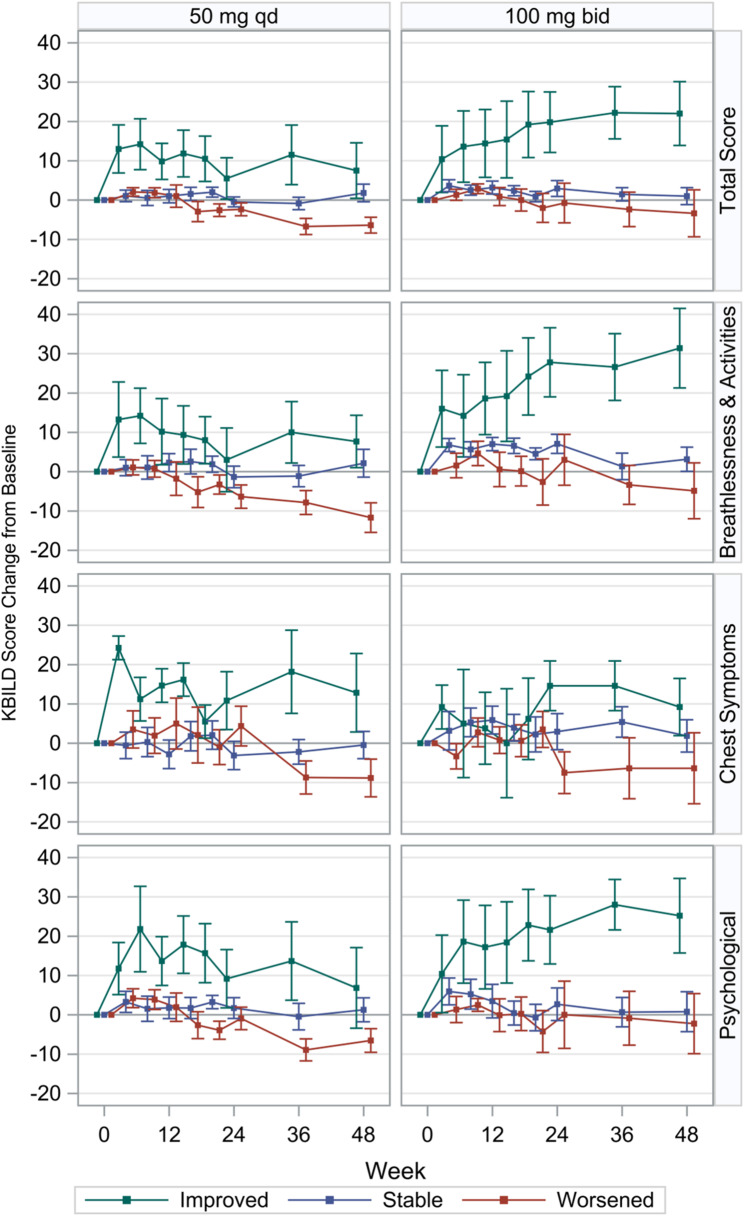
KBILD change from baseline by category of change in QLF (% of whole lung). bid = two times per day; KBILD = King’s Brief Interstitial Lung Disease questionnaire; qd = once per day; QLF = quantitative lung fibrosis

Mean changes over time in KBILD total and domain scores by QLF improvement for each dose group are shown in Fig. 4. Patients in the 100-mg bid group with improved QLF showed an increase in KBILD total scores over time. Both the breathlessness and activities domain and psychological domain contributed to this improvement. Differences are apparent as early as week 8 and maintained through week 48 in the 100-mg bid group. At week 48, the mean change from baseline for the patients in the 100-mg bid group with improved QLF was 22.0 (SD 18.6) for KBILD total score and 31.4 (SD 23.0) for breathlessness and activities, 9.2 (SD 16.7) for chest symptoms, and 25.2 (SD 21.6) for psychological factors, all of which were greater than the MCID of 5 for KBILD.

## Discussion

We report the effect of the inhaled therapy AP01 in a phase 1b study of two dosing regimens on changes in the extent of fibrosis and lung volumes obtained from quantitative evaluation of HRCT and in QOL scores in patients with IPF. With the association of structural and functional changes having been previously reported [5], the post-hoc analyses presented here show correlation of the structural improvements quantified in the QLF scores with symptomatic improvements assessed by PRO measures. As measured by QLF score, the extent of fibrosis stabilised in some patients after treatment with AP01 and decreased in a few. Additional data supported the observed changes in fibrosis. Increases in fibrosis, as measured by HRCT, were smaller in the 100-mg bid group than in the 50-mg qd group.

Quantitative evaluation of HRCT has been incorporated as an outcome measure in several recent trials of antifibrotic agents in IPF. Patients treated with nintedanib or pamrevlumab, an investigational antifibrotic agent, demonstrated smaller increases in fibrosis as measured by HRCT than those treated with a placebo control. Lancaster et al. reported an increase from baseline in the adjusted mean QLF score of 21.7 mL for nintedanib, compared with 37.3 mL for the placebo group (Spearman correlation − 0.63) [11]. Richeldi et al. reported an increase of 24.5 mL for pamrevlumab, compared with 86.7 mL for the placebo group (Spearman correlation − 0.45) [10]. The increase of 25.8 mL in the AP01 50-mg qd group in this study is comparable to the active agents in Lancaster et al. and Richeldi et al. and smaller than their reported adjusted placebo means [10, 11]. Additionally, a decrease in mean QLF score of 29.5 mL in the 100-mg bid dose group was observed in this study, suggesting a reduction in fibrosis that was not reported for nintedanib or pamrevlumab [12]. 

Five patients (16%) on the higher 100-mg bid dose with an improvement in QLF scores at 24 weeks showed an early and sustained improvement in the validated KBILD score, which exceeded the MCID. To our knowledge, this is the first time an improvement in QLF scores has been significantly associated with an improvement in QOL.

In addition to the previously reported correlation between change in QLF score (mL) and change in FVC at 24 weeks in the 100-mg AP01 group [5], the analyses reported here show early structural changes at 24 weeks indicated by QLF score aligned with the symptomatic improvement reported at Week 48. Modest correlations were observed for the 50-mg qd group. The magnitude of improvement in QLF revealed by HRCT and in symptomatic changes revealed by KBILD scores was pronounced in the 100-mg bid group.

Our study included several limitations, which may impact the interpretation of the results. These limitations include the relatively small sample size and the absence of a placebo control. In addition, some post-baseline HRCT scans were conducted earlier than scheduled because of early terminations or later than planned because of 2020–2021 COVID-19 pandemic restrictions. Some post-baseline studies were never completed. Finally, site-determined evidence for UIP may have been heterogeneous. Despite these limitations, the stabilization or even decrease in the extent of fibrosis in some patients was encouraging, as was the inverse correlation between QLF score and FVC, consistent with earlier studies. Together, these results indicate that further investigation of AP01 is merited and support the continued use of quantitative evaluation of HRCT in IPF clinical trials.

## Conclusions

The quantitative scores from HRCT support selection of the 100-mg bid clinical dose for further study. After treatment with AP01 in this phase 1b study of patients with IPF, the extent of fibrosis as measured by HRCT stabilised in some patients and decreased in a few. The 100-mg bid group showed smaller increases in fibrosis on average than the 50-mg qd group. Composition data supported the observed changes in fibrotic progression. Patients in the 100-mg bid group with improved QLF scores showed improvement in KBILD scores, with the total scores improving more than 20 points from baseline by week 48. Associations between changes in FVC, KBILD, and QLF confirm the functional, symptomatic, and structural changes in IPF patients. Furthermore, they support the 100-mg bid dose moving forward into the next AP01 study.

With continued follow-up of HRCT scans, our data suggest that the association between the QLF imaging biomarker and supportive physiological and QOL measures in the 100-mg bid dose may provide additional supportive data on dose-ranging for future studies. The results appear to be encouraging in the context of results from other recent trials of antifibrotic agents, such that further investigation of AP01 is merited.

## Supplementary Information

Supplementary Material 1.

## Data Availability

The datasets used and/or analysed during the current study are available from the corresponding author on reasonable request.

## Abbreviations

AP01: Inhaled pirfenidone
ATS: American Thoracic Society
bid: Twice a day
COVID-19: Coronavirus disease 2019
ERS: European Respiratory Society
FVC: Forced vital capacity
HRCT: High-resolution computed tomography
IPF: Idiopathic pulmonary fibrosis
KBILD: The King’s Brief Interstitial Lung Disease
LCQ: Leicester Cough Questionnaire
MCID: Minimally clinically important difference
PRO: Patient-reported outcome
QCT: Quantitative computed tomography
QGG: Quantitative ground glass
QHC: Quantitative honeycomb
QLF: Quantitative lung fibrosis
QOL: Quality of life
qd: Once daily
TLC: Total lung capacity
UIP: Usual interstitial pneumonia
