# Supplementary material for: Improved quality of life, reduced quantitative lung fibrosis in a trial of inhaled pirfenidone for idiopathic pulmonary fibrosis
Source: BMC Pulm Med. 2026 Mar 23;26:202. doi: 10.1186/s12890-026-04234-x (PMC13130782; doi:10.1186/s12890-026-04234-x)
Supplement: Supplementary file 1 — Supplementary Material 1. [file 12890_2026_4234_MOESM1_ESM.pdf]

Supplemental Table 1. Ethics Committees/Institutional Review Boards and approval IDs/numbers for clinical trial ACTRN 12618001838202

**Country**	**Site**	**Ethics Committee(s) / Institutional Review Board(s)**	**Number/ID of approval**
Australia	AU01	Bellberry Limited HREC 129 Glen Osmond Rd, Eastwood, South Australia, 5063, Australia	HREC2018-10-899-AA
	AU0	Hunter New England Human Research Ethics Committee The Lodge, Rankin Park Campus, Lookout Road, New South Wales, 2305, Australia	HREC/18/HNE/315
	AU04		
	AU05	The Prince Charles Hospital 627 Rode Road Chermside, Brisbane, QLD, Australia, 4032	
	AU06	Hunter New England Research Ethics and Governance Office Locked Bag #1 New Lambton, New South Wales, 2305, Australia	
	AU08		
	AU12		
	AU09	Bellberry Limited HREC 123 Glen Osmond Road, Eastwood, South Australia, 5063, Australia	HREC2018-10-899-AA
	AU10	Central Adelaide Local Health Network Human Research Ethics Committee Level 3, Roma Mitchel House, North Terrace, Adelaide, South Australia, 5000 Australia	HREC/19/CALHN/342
	AU15	Bellberry Human Research Ethics Committee 123 Glen Osmond Road, Eastwood, South Australia, 5063, Australia	HREC2018-10-899-AA
Czech Republic	CZ01	Eticka komise IKEM a Thomayerova nemocnice Thomayerova nemocnice, Videnska 800 ,Praha 4, Czech Republic, 140 59 Eticka komise FN Olomouc, I.P. Pavlova 6, Olomouc, Czech Republic, 779 00	12388/19
	CZ02	Eticka komise FN Olomouc, I.P. Pavlova 6, Olomouc, Czech Republic, 779 00	1/19 MEK 1
Great Britan	GB01	Yorkshire and the Humber — Leeds East Research Ethics Committee HRA Office, Holland Drive, Newcastle upon Tyne, NE2 4NQ	REC reference: 19/YH/0093; IRAS ID: 255567
	GB03<br>GB04<br>GB06<br>GB07	MEC-U, Koekoekslaan 1, 3435 CM, Nieuwegein, The Netherlands	
The Netherlands	NL01		R18.089/ATLAS study*
	NL02		
New Zealand	NZ01	Health and Disability Ethics Committees, Ministry of Health 133 Molesworth street, Wellington, 6011, New Zealand	18/CEN/246/
	NZ02		
	NZ03		
Poland	PL02	Komisja Bioetyczna przy Uniwersytecie Medycznym w Lodzi Pl. Hallera 1B, 90-647 Lodz, Poland	RNN/28/19/KE*
	PL03		
	PL04		
	PL06		

* Central ethics committee submission only; there were no local ethics committees.
